# Transient Global Amnesia as a Rare Consequence of Diagnostic Cerebral Angiography at an Ambulatory Neurosurgery Center: A Case Report

**DOI:** 10.7759/cureus.105368

**Published:** 2026-03-17

**Authors:** Lukasz Przepiorka, Jaims Lim, Rosalind Lai, Adnan H Siddiqui, Elad I Levy

**Affiliations:** 1 Neurosurgery, Jacobs School of Medicine and Biomedical Sciences, University at Buffalo, Buffalo, USA; 2 Neurosurgery, Gates Vascular Institute, Buffalo, USA; 3 Canon Stroke and Vascular Research Center, University at Buffalo, Buffalo, USA; 4 Research, Jacobs Institute, Buffalo, USA; 5 Radiology, Jacobs School of Medicine and Biomedical Sciences, University at Buffalo, Buffalo, USA

**Keywords:** ambulatory neurosurgery center, cerebral angiography, "conscious sedation"[mesh]), digital subtraction angiography(dsa), postprocedural complications, transient global amnesia

## Abstract

Although invasive, digital subtraction angiography (DSA) is considered a relatively safe procedure and remains the gold standard for cerebrovascular imaging. Transient global amnesia (TGA) is a self-limited episode of sudden-onset anterograde amnesia that resolves spontaneously within 24 hours. We report a rare occurrence of TGA after diagnostic DSA. A 62-year-old man presented for an angiogram performed under conscious sedation for the diagnosis of a suspected cavernous internal carotid artery aneurysm. Although the DSA revealed normal anatomy, the patient experienced self-resolving memory impairment immediately after the procedure. To date, only a few such cases have been reported, and this is the first documented instance that occurred at an ambulatory neurosurgery center. This case highlights that rare phenomena, such as transient global amnesia, can occur after cerebral angiography, even with brief radiation exposure and a short procedure duration. Recognizing such events is particularly important as neurosurgical care continues to transition from hospitals to outpatient centers, serving as a key safeguard against the shorter observation periods at these ambulatory facilities.

## Introduction

Digital subtraction angiography (DSA) is the 'gold standard' of cerebrovascular imaging [1]. Although invasive, the procedure is considered relatively safe, with overall complication rates ranging from 2.6% to 5% [2,3]. Transient global amnesia (TGA) is a self-limited episode of sudden-onset anterograde amnesia that resolves spontaneously within 24 hours. The diagnosis of TGA can be made through observation by a reliable witness and the exclusion of other potential causes, such as focal neurological deficits or seizures [4,5]. Although the exact pathophysiological mechanism of TGA remains unclear, precipitating factors, including psychological stress or medical procedures, are often reported [5,6]. Here we describe a patient who experienced this rare condition following DSA performed at an ambulatory neurosurgery center, which, to our knowledge, has not previously been reported.

## Case presentation

A 62-year-old man presented to an ambulatory neurosurgery center for diagnostic DSA after the incidental detection of a possible medially projecting cavernous left internal carotid artery (ICA) aneurysm. Magnetic resonance angiography (MRA) had been performed for the evaluation of a sudden-onset headache that had developed after the use of a phosphodiesterase inhibitor. Additionally, the patient had reported a lingering dull headache lasting 10 days, accompanied by sneezing. His medical history was significant for prediabetes, mixed hyperlipidemia, and essential hypertension. Given the abrupt onset of headache, MRA was conducted to screen for an aneurysm and revealed a suspected left ICA aneurysm, prompting the recommendation for a diagnostic angiogram.

The DSA was performed using a right radial artery approach. A local anesthetic composed of bupivacaine was given, followed by an infusion of 2.5 mg of verapamil and 2,500 units of heparin through the radial 5-French sheath (Glidesheath™ Slender, Terumo, Somerset, NJ, USA). Conscious sedation was induced with 1 mg of midazolam and 50 µg of fentanyl in this 102 kg man. The left and right common carotid arteries, left ICA, left external carotid artery, and right vertebral artery were selectively catheterized. In addition, a three-dimensional left ICA angiogram was performed.

**Figure 1:**
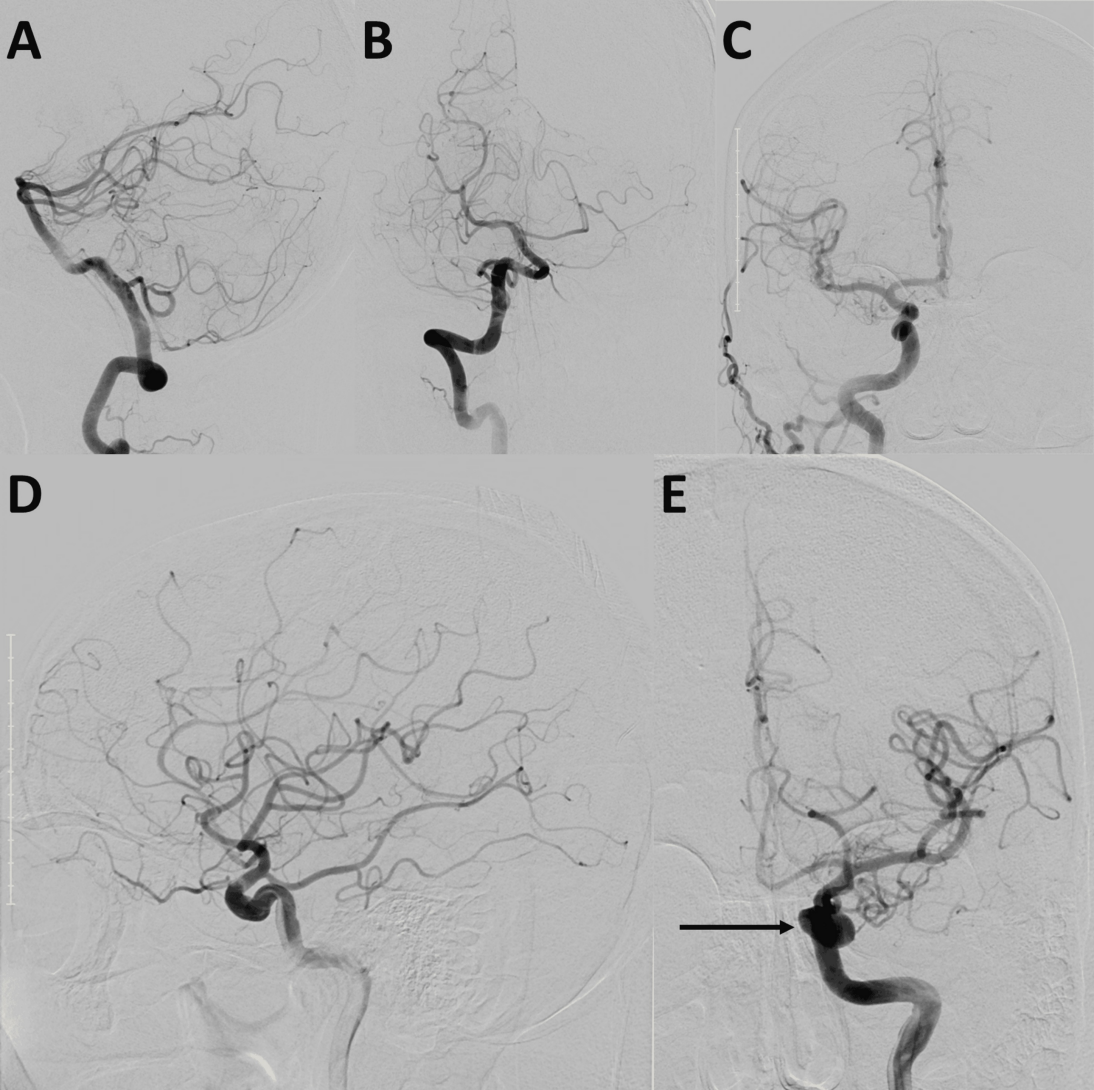
(A-E) Digital subtraction angiography images of the presented case. (A) Lateral and (B) anterior views of the normal anatomy of the posterior circulation; (C) anterior view of right common carotid artery injection without abnormalities; (D) lateral and (E) anterior views of the left internal carotid injection, significant for the normal anatomical dilation of the left cavernous segment (arrow).

The DSA revealed a normal anatomical dilation of the left cavernous ICA, with no aneurysms or other pathologies present (Figure 1A-E). The total time for the diagnostic angiogram was 6 minutes from the time of needle puncture until the catheter was fully removed. The total fluoroscopy time was 3.4 minutes, with a radiation dose of 491.2 mGy. An iohexol contrast agent was administered during the procedure. The right radial arteriotomy site was secured using a Vasc Band™ closure device (Teleflex, Wayne, PA, USA).

Postprocedurally, the patient exhibited isolated short-term and long-term memory deficits, characterized by repetitive questioning, without any additional neurological deficits. These memory impairments persisted for 4 to 6 hours before resolving, during which the patient was closely observed. Throughout this time, he remained fully alert and hemodynamically stable, making a prolonged sedative effect unlikely. At his follow-up visit two weeks later, the patient confirmed resolution of headaches, no further confusion, and a healed radial access site.

## Discussion

Prevalence of TGA among patients undergoing DSA

TGA is a rare, but known, complication of DSA; in a prospective evaluation of 2,899 DSA procedures, the rate of TGA was 0.2% [7]. Furthermore, a limited number of case reports or case series have documented TGA after DSA [8-13]. In the thorough evaluation by Quinette et al., a precipitating event, including anxiety or exhaustion, was found in up to 89% of TGA cases [5]. Among all procedure-related TGA occurrences in a study by Jeong et al., DSA was the most frequent precipitating event [6].

Possible mechanisms linking TGA and DSA

Early reports have speculated on potential TGA causes, including ischemia-particularly due to vasospasm. Jackson et al. reported a series of patients with arterial spasm and subsequent TGA, which they attributed to a faulty contrast material warming cabinet, resulting in the injection of contrast material with higher than usual temperatures [12]. Juni et al. found spasm of the left vertebral and basilar arteries during DSA performed for the evaluation of subarachnoid hemorrhage, after which the patient developed TGA [13]. Repeat imaging, including computed tomography and a three-month DSA, showed no abnormalities. More recent studies have drifted away from describing arterial ischemia as the cause of TGA [14]. Instead, some researchers have found a strong association between internal jugular vein valve incompetence and TGA, although their exact pathogenic relationship is yet to be discovered [15,16]. In the context of the present case, DSA may have been the triggering event.

The other potential explanation for the observed deficit is the side effect of the administered analgesic medications. Midazolam acts on gamma-aminobutyric acid receptors through which it exerts its effect of anterograde amnesia. As such, it could have been the causative agent of the neurological deficit, with its half-life of 1.5 to 2.5 hours. Fentanyl is an opioid agonist and has a half-life of 3 to 7 hours. Activation of its receptors produces analgesia and increases dopamine in the reward areas of the brain, causing the stereotypical effects of exhilaration and relaxation.

Apart from these administered medications, the possibility of iohexol-induced TGA cannot be excluded. However, in case reports of angiography-related complications, contrast-induced encephalopathy, which has a different presentation, has been described [17].

Interpretation

Because the diagnosis of TGA can be made on the basis of clinical presentation and neurological examination, it is vital to recognize it as a rare complication of DSA. This is particularly crucial in the context of transitioning neurosurgical care to ambulatory centers, where the shorter observation period demands heightened vigilance to ensure patient safety [18-20]. The potential value of additional evaluation, particularly brain imaging, lies in excluding alternative diagnoses [4].

## Conclusions

This study serves as a reminder of the possibility of rare phenomena, such as TGA, after cerebral angiography. These events can occur despite the short radiation exposure and duration of the procedure and should be differentiated from other conditions with a similar presentation that may have consequences that are more serious and lack a self-limiting course. Recognizing such rare events is particularly important as neurosurgical care transitions from hospitals to ambulatory centers. This transition will require heightened clinical awareness to facilitate prompt evaluation and avoid unnecessary interventions.
